# The Efficient Coding of Speech: Cross-Linguistic Differences

**DOI:** 10.1371/journal.pone.0148861

**Published:** 2016-02-22

**Authors:** Ramon Guevara Erra, Judit Gervain

**Affiliations:** Laboratoire Psychologie de la Perception, CNRS and Université Paris Descartes, Sorbonne Paris Cité, Paris, France; Max Planck Institute for Human Cognitive and Brain Sciences, GERMANY

## Abstract

Neural coding in the auditory system has been shown to obey the principle of efficient neural coding. The statistical properties of speech appear to be particularly well matched to the auditory neural code. However, only English has so far been analyzed from an efficient coding perspective. It thus remains unknown whether such an approach is able to capture differences between the sound patterns of different languages. Here, we use independent component analysis to derive information theoretically optimal, non-redundant codes (filter populations) for seven typologically distinct languages (Dutch, English, Japanese, Marathi, Polish, Spanish and Turkish) and relate the statistical properties of these filter populations to documented differences in the speech rhythms (Analysis 1) and consonant inventories (Analysis 2) of these languages. We show that consonant class membership plays a particularly important role in shaping the statistical structure of speech in different languages, suggesting that acoustic transience, a property that discriminates consonant classes from one another, is highly relevant for efficient coding.

## Introduction

Increasing evidence suggests that neural representations in the auditory system follow the principles of efficient neural coding [1], an information theoretical principle known to underlie neural coding in several perceptual systems [2]. The theory of efficient neural coding holds that the sensory systems have evolved to encode environmental signals in an information theoretically optimal way, representing the greatest amount of information at the lowest possible cost [3,4]. To achieve this information theoretical optimum, the sensory systems need to capture the underlying statistical structure of environmental signals [5,6]. Indeed, it has been shown that mathematically derived efficient codes for different natural stimuli closely resemble neural response functions measured in the visual [7,8] and, more recently, in the auditory systems [1,9,10].

A better understanding of the statistical structure of natural stimuli is crucial for the assessment of the efficient coding theory and for a more fine-grained description of neural coding in general. Recent work [1,10,11] suggests that the statistical structure of speech is particularly similar to the auditory neural code. However, only one language, English, has so far been investigated. It thus remains unknown how much variation there is in the statistical structures of different languages. Languages of the world exhibit considerable differences in their sound patterns, e.g. in their speech rhythm, phoneme repertoire, syllable structure etc. To what extent and how these linguistic differences are reflected in the overall statistical structure of a language has not yet been explored. The first objective of the current study is to address this question. Specifically, we will use independent component analysis (ICA) to derive information theoretically optimal, non-redundant codes (filter populations) for seven typologically distinct languages (Dutch, English, Japanese, Marathi, Polish, Spanish and Turkish) and relate the statistical properties of these filter populations to documented differences in the speech rhythms (Analysis 1) and phoneme repertoires (Analysis 2) of these languages.

### The efficient coding of natural auditory stimuli

Paralleling previous work in vision research [2,7], an increasing number of studies has recently investigated whether, and if yes, how the statistical structure of natural sound stimuli might be reflected in the auditory neural code [1,10,12] and how they might be related to sound percepts in humans [11,13–15].

Of relevance for the current study is the finding [1,10] that physiologically measured auditory nerve responses in mammals [16–18] appear to best match the statistical properties of two specific sound classes. The first is a mixture of transient environmental sounds (e.g. breaking branches, cracking ice, dripping water etc.) and animal vocalizations. Transient environmental sounds, which are short, non-harmonic, broad-band signals, can be statistically characterized by a population of wavelet-like filters, localized both in frequency and time, whereas mammalian vocalizations, which are long, harmonic, narrow-band sounds, are best captured by Fourier-like filters, localized in frequency, but not in time. The mathematically derived efficient filters for a mixture of these two types of sounds match remarkably well the reverse correlation filters obtained from electrophysiologically measured auditory nerve responses. The second sound class for which this match is particularly strong is speech. Indeed, speech is a mix of harmonic and transient sounds and its theoretically derived efficient filter population is in between the wavelet-like filters for environmental sounds and the Fourier-like filters for animal vocalizations.

One way to characterize and numerically compare the filter populations for different sounds is to calculate the regression between the center frequency and the sharpness (center frequency divided by the bandwidth) of each filter within a population. The slopes of the resulting regression lines can then be compared across different filter populations. Consistently with the observation that the average power spectrum of speech, music and many other sounds is approximately 1/*f* [19], following a power law distribution, sharpness increased with center frequency for all three sound classes [10]. However, the slopes of the regression lines differed. The filter population for animal vocalizations had the steepest regression slope, as in this Fourier-like filter population the bandwidth of filters was almost constant and didn’t scale very strongly with center frequency. By contrast, filters for environmental sounds had bandwidths that increased with increasing center frequency. The slope of the regression line was therefore less steep for this filter population. The slope for speech fell in between the other two slopes.

These results are consistent with the idea [10] that speech is a mix of transient and harmonic sounds, possibly because speech has evolved to recruit the two already existing neural codes, i.e. wavelet-like filters for environmental sounds and Fourier-like filters for vocalizations. Indeed, speech sounds greatly differ in harmonicity and transience. Stop consonants, for instance, are typically transient and non-harmonic, resembling environmental sounds, whereas vowels are longer and harmonic, like vocalizations. One prediction of this proposal, therefore, is that languages differing in the relative proportion of vowels and consonants should have efficient filter populations with different slopes.

Following up on this hypothesis, the efficient filter populations for different vowel and consonant subclasses have recently been investigated [20]. It has been found that efficient filter populations for different sound classes do indeed exhibit different slopes. Importantly, however, the main difference is found not between vowels and consonants, but between different consonant sub-classes. Specifically, different vowel classes (e.g. back vs. front; high vs. low) all have similar slopes, which closely match the slope of the filter population for speech in general [10]. By contrast, efficient filters for consonant classes show important variations in their slopes, with stop consonants (e.g. /b/, /t/, /p/ etc.) having the lowest slopes, close to that of transient environmental sounds, affricates (e.g. /tʃ/ etc.) having medium slopes, close to that of vowels and of speech in general, and fricatives (e.g. /s/, /f/ etc.) as well as nasals having steep slopes, similar to that of animal vocalizations. These results thus diverge from the original proposal [10], which suggested that harmonicity and transience both play a role in determining the statistical structure of speech sounds, rendering vowels similar to vocalizations, and consonants to environmental sounds. This more recent study [20] suggests instead that since consonants are not harmonic and consonant sub-classes differ greatly in their acoustic transience, it is solely this latter that accounts for differences between sound classes, and harmonicity or bandwidth play a less important role.

The second objective of the current study is, therefore, to explore further which acoustic properties of speech underlie differences in the efficient codes for different languages, if such cross-linguistic differences can indeed be found. The respective roles of transience, harmonicity and bandwidth in determining the statistical structure of different languages and hence their efficient codes can be tested based on their phonological and perceptual correlates. Harmonicity correlates with the vowel/consonant distinction. The relative proportion of vowels and consonants in the speech signal is in turn related to the notion of speech rhythm. In fact, the relative proportion of vowels and consonants and the variability in vocalic and consonantal intervals in the speech signal are well-established operational measures of speech rhythm [21]. Therefore, deriving efficient codes for languages with different speech rhythms allows us to investigate the role of harmonicity. If harmonicity plays a role, as initially suggested [10], then we expect the efficient codes for languages to vary as a function of their speech rhythm, a well-documented factor of cross-linguistic variability.

If, by contrast, only transience is decisive [20], then vowels are less relevant for determining the slope of the sharpness regression line, and differences in the types of consonants found in a language are expected to underlie cross-linguistic differences in derived efficient codes. Languages of the world differ considerably in their consonant inventories. Therefore, by testing whether cross-linguistic differences between the efficient filters of different languages align with differences in speech rhythm, in consonant inventory or in both, we can determine what acoustic cues contribute most to cross-linguistic differences in the statistical structure of speech.

### Cross-linguistic variations in speech rhythm and phoneme repertoire

The languages of the world show systematic variation in their speech rhythms and phoneme repertoires. Linguists had traditionally categorized languages into three rhythmic classes: syllable-timed languages, such as Spanish or Italian, stress-timed languages, like English or Dutch, and mora-timed languages, like Japanese and Tamil. This classification was operationalized [21] using three measures for describing and quantifying language rhythm: %V, i.e. the relative length of vocalic space in the speech signal, ΔV, i.e. the variability in the length of vocalic spaces, and ΔC, i.e. the variability in the length of consonant clusters. The authors measured these three properties in eight languages, English, Dutch, Polish, Catalan, Italian, Spanish, French and Japanese, and found that they cluster into three groups similar to the original classification when plotted in the two-dimensional spaces defined by any two of these measures. The measure %V appeared to correlate particularly well with previous classifications. This operational definition, which provides a continuous measure, has the advantage of yielding a quantitative basis for the original classification, yet allowing languages with mixed or ambiguous rhythm to be accommodated.

The seven languages used in the current study have the following rhythmic properties. Dutch, English and Polish are traditionally described as stress-timed languages, and have relatively low %V values [21], Spanish, Turkish and Marathi are syllable-timed languages, with medium to high %V values [21–24], and Japanese is a mora-timed language, with very high %V values [21]. (Note that quantitative definitions of speech rhythm other than %V, ΔV and ΔC have been proposed in the literature [25–30]. Here we will be using the original %V, ΔV and ΔC measures, because the seven languages we analyze all have published %V, ΔV and ΔC values, whereas some lack the other metrics. Furthermore, our analyses are based on the same sound files as the published %V, ΔV and ΔC measures, ensuring full comparability between our study and the previous rhythm measures.)

Speech rhythm is critical in every aspect of speech perception from the earliest prenatal experience through language acquisition to adult language comprehension. Indeed, speech is first experienced in the womb as a low-pass filtered signal, transmitting only global speech prosody and rhythm, but not individual sounds. Accordingly, newborns are able to recognize their native language on the basis of its rhythm [31] and can discriminate languages they have never heard before if those are rhythmically different [32–34]. Adults also use speech perception mechanisms optimized for the rhythmic characteristics of their native language [35,36], and are better able to maintain intelligibility under a wide set of circumstances (speech in noise, accelerated speech etc.) for non-native languages that resemble the rhythm of their native language than for those that are rhythmically different [37].

Importantly for the purposes of the current study, the above definition of rhythm relies on the vowel/consonant distinction, but not on the precise identity of specific vowels and consonants. This predicts that languages are perceived as having different rhythms as long as the relative timing of vocalic and consonantal spaces are preserved, even if the identities of the individual phonemes are suppressed. These predictions have been confirmed [38] by showing that adults were able to discriminate rhythmically different languages, even if the signal was resynthesized replacing all vowels by /a/ and all consonants within a consonantal class by a representative of that class (fricatives by /s/, liquids by /l/, occlusives by /t/, nasals by /n/, and glides by /j/; the “saltanaj” transformation) or all vowels by /a/ and all consonants by /s/ (the “sasasa” transformation). Thus language rhythm is not sensitive to vowel and consonant identity or consonant class. However, language discrimination failed if all segments were replaced by /a/, i.e. if the consonant/vowel distinction was abolished.

Nevertheless, languages also differ in their speech sounds. The sizes of phoneme inventories in languages vary from a dozen sounds to well over a hundred sounds. Consonant inventories also show large variations, from as few as six consonants up to more than a hundred [39]. The size principle argues that languages with smaller consonant inventories mainly have consonants that are phonetically, phonologically or articulatorily simpler, e.g. oral stop consonants, while languages with larger inventories have more complex consonant classes [40]. For instance, Rotokas, a language spoken in Papua New Guinea, only has 6 consonants, /p/, /t/, /k/, /b/, /d/ and /g/, which are exactly the oral stop consonants. Unlike in the case of rhythm, consonant class inventory is preserved under a “saltanaj”-like transformation, but not under a “sasasa”-like transformation. The languages of the current study have the following consonant class inventories [39,40]. English has an average-sized consonant inventory, in which all major consonant classes, i.e. stops, laterals, glides, affricates, fricatives and nasals, are represented. Stop consonants constitute the most frequent class, fricatives are the second, laterals and glides together come next, followed by nasals. Affricates are the least frequent. In a speech corpus of adult-directed conversational English, stops made up about 20% of their corpus, fricatives accounted for approx. 16%, laterals and glides 11%, nasals 10%, and affricates 1% [41]. Dutch and Turkish also has an average-sized consonant inventory, with consonants in all major consonant classes [42,43]. The consonant inventory of Spanish is also average-sized, although somewhat smaller than that of English, Dutch and Turkish, as affricates are lacking and fricatives are fewer in number [44]. Polish has a large phoneme inventory, with dental stops, fricatives, nasals and affricates, which are not present in most of the other languages. The consonant inventory of Marathi is also large, with dental and retroflex stops and nasals not present in the other languages, although it has fewer fricatives and no affricates [45]. Japanese, by contrast, has a relatively small consonant inventory, with no affricates and fewer fricatives than in the other languages.

In sum, the current study seeks to test whether the statistical structure of speech in different languages shows cross-linguistic differences, and if yes, to what phonological and acoustic properties of speech these might be related to. Specifically, we test whether cross-linguistic differences may be related to the different proportions of vocalic and consonantal intervals in the speech signals of different languages, i.e. a correlate of speech rhythm, and/or whether they may be related to differences in phoneme, in particular consonant, repertoires across the world’s languages. In the former case, the relevant physical properties of the acoustic signal are harmonicity, bandwidth and transience; in the latter case, only transience plays a role. Note that the two hypotheses are not mutually exclusive: speech rhythm and phoneme inventories show correlations across the world’s languages [46,47]. It is, therefore, possible that the properties of efficient populations are related to both of these linguistic features. However, our analyses will be able to shed light on their respective contributions to the statistical structure of the speech signal.

## Analysis 1

**Fig 1:**
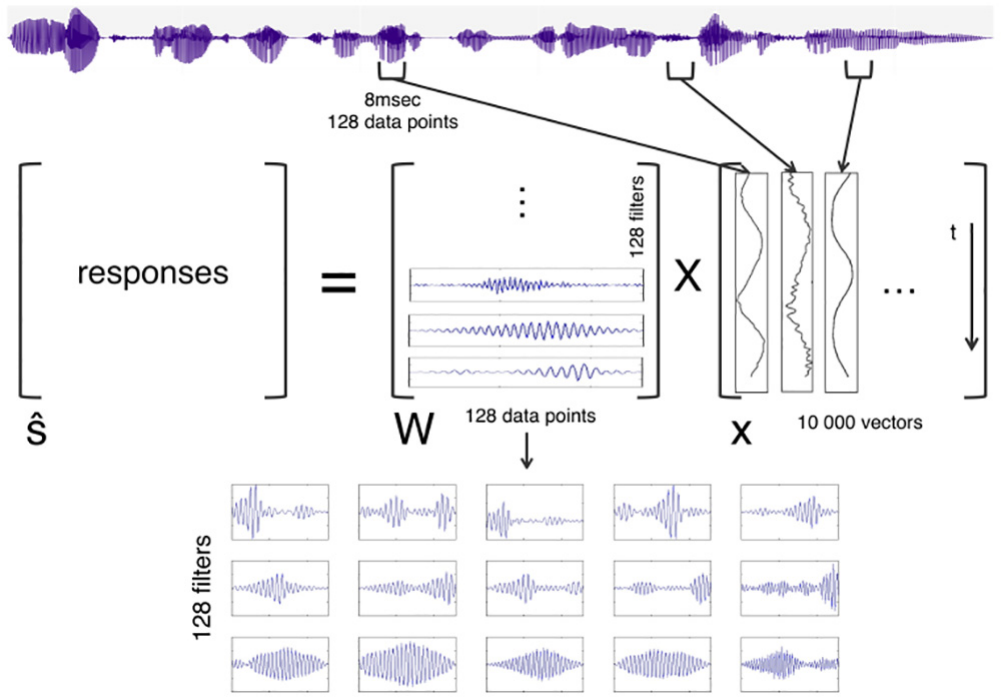
Algorithm. The ICA algorithm used in the current study.

In Analysis 1, we tested whether theoretically derived efficient filters for speech in seven rhythmically different languages correlated with existing acoustic measures of speech rhythm. We used a generalized independent component analysis (ICA) algorithm [10,48] to achieve an information theoretical optimum, i.e. the encoding of the speech signal with a set of independent filters that capture the statistical structure of the input at the lowest cost, i.e. with no redundancy. In this framework, it is assumed that the linear response of the auditory system, ${\hat{s}}_{i}\left(t\right)=\sum_{\tau =0}^{N-1}w_{i}\left(t-\tau \right)x\left(\tau \right)$, is given by the convolution of a set of filters *w*_*i*_(*t*) with the signal *x*(*t*) of length *N* (in matrix form, ŝ = Wx). Efficient encoding is achieved if the set of filters is such that the statistical dependence of the responses ŝ is minimized. As the input to this analysis (Fig 1), we used speech samples from Dutch, English, Japanese, Marathi, Polish, Spanish and Turkish. These languages were chosen because they are geographically and historically unrelated, represent typologically different phonological, morphological and syntactic structures and, most importantly, belong to different rhythmic classes, as previously described [21–23]. To maximize comparability between earlier prosodic measures and the current analysis, we used the same sound files as the previous prosodic studies [21–23].

### Stimuli

The speech samples consisted of sentences recorded by female native speakers in Dutch, English, Japanese, Marathi, Polish, Spanish and Turkish, all obtained from previous studies on the prosodic and rhythmic properties of these languages [Dutch, English, Japanese, Polish [21]; Marathi, Turkish [22]; Spanish [23]]. For each language, the dataset consisted of simple declarative, news-like sentences ranging between 15 and 21 syllables in length, recorded each by four different female native speakers at a sampling rate of 16 kHz. The sentences in the different languages were roughly matched for meaning. The input dataset to the ICA algorithm was then constructed by randomly selecting 10000 different 128-datapoint-long segments [corresponding roughly to 8msec chunks at the current sampling rate) from the speech samples for each language.

As a basis for comparison with rhythmic measures, we used %V (as reported in [21] for English, Japanese, Dutch and Polish, in [22] for Marathi and Turkish and [23,24] for Spanish. We chose to use %V rather than other existing measures [25,26,29], because we had access to the sound files used to obtain the above cited %V measures. Furthermore, measures of %V have been linked to language discrimination and speech perception abilities in newborns, infants and adults [21,24,34,49,50], constituting solid evidence in favor of the psycholinguistic validity of this measure.

### Algorithm

According to the efficient coding hypothesis, redundancy between information processing channels is reduced in sensory systems so as to maximize capacity for each channel (in the nervous system, a code that maximize channel capacity is a key design constraint, given the high metabolic cost of spiking neurons). In other words, efficient coding is achieved if processing channels are as independent as possible from each other (so, minimizing redundancy). In this view, the transformation from input to output of a sensory system is such that the output channels are maximally independent. From a signal processing point of view, early stages in auditory processing can be modelled as a transformation from incoming auditory signals (input) to neural activity encoding auditory information (output). The passing from input to output in the auditory system has been successfully modelled as a linear transformation [16,17] effected by appropriate filters acting on auditory signals. According to the efficient coding hypothesis, early stages in the auditory system can be modelled as a linear transformation that minimizes mutual information between output channels. This is of course an approximation valid within a limited dynamical range, where non-linear effects of the early auditory system can be neglected. Since we are interested in the filters that act on the auditory signal, we need to solve an inverse problem (signal deconvolution): what is the linear transformation (whose matrix is formed by those filters) such that when applied to a given set of auditory signals gives statistical independence between the output channels?

This problem is typically solved in signal analysis by applying independent component analysis (ICA), an algorithm for blind signal separation and signal deconvolution. ICA assumes that a mixture of independent sources results in the registered signals. The task of the algorithm is to find the sources and the mixing matrix. More formally, given a linear combination of sources *s* (*x* = *As*) and assuming that the sources are statistically independent, ICA reconstructs the sources as ŝ = Wx. ICA has been successfully used in the context of the efficient coding hypothesis, for both natural images [51] and auditory signals [10].

We therefore used ICA for blind signal deconvolution of the speech time series, following the specifications of the ICA algorithm in [10]. A sample of 10 000 speech time-series segments per language were introduced as vector columns in *x*, each vector consisting of 128 samples. The number of samples was chosen to be 128 for three reasons. First, given the sampling rate of the sound files, 128 samples roughly correspond to time series of about 8msec in duration. This window size is appropriate to capture (sub)phonemic information, i.e. the type of information relevant for language rhythm and the discrimination of consonants, vowels and their different sub-classes. Second, this window size was used in previous work [10,20], allowing for easier comparison across studies. Third, this window size also ensured easy and rapid computation in our ICA algorithm. The filters were the rows of the unmixing matrix *W*. The specific ICA algorithm used was RUNICA, which uses the logistic infomax ICA algorithm [51] with the natural gradient feature [52].

### Results

**Fig 2:**
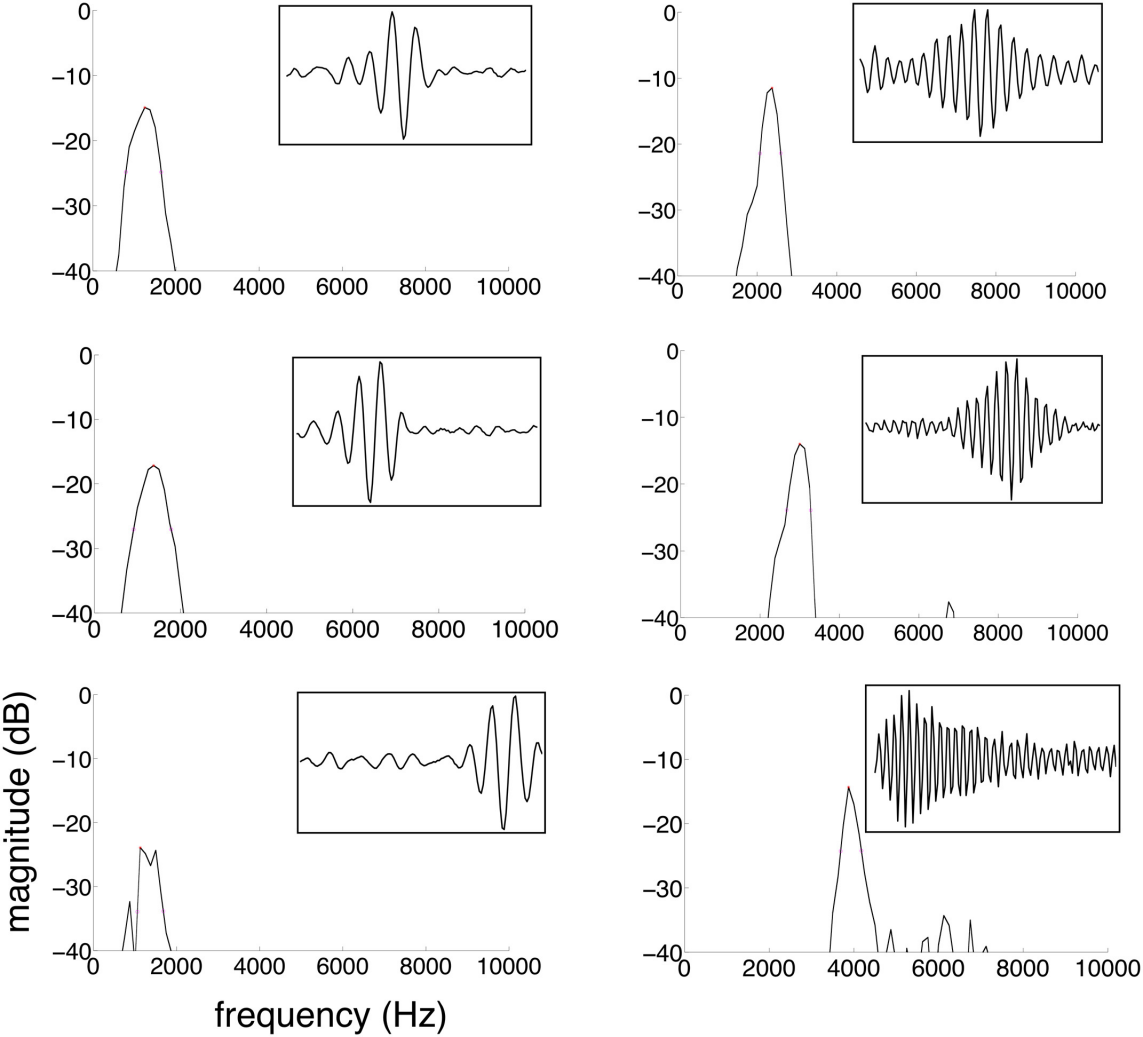
Filter populations. Representative examples of filters (insets) and their spectra obtained for English.

The ICA algorithm generated a set of filters (unmixing matrix), each defined by 128, independently varying points with no a priori constraints on filter shape. Examples of the filters obtained and their spectra are shown in Fig 2. The filters obtained have a gammatone shape, with an amplitude envelope relatively well localized in time. These filter shapes are similar to those obtained in other studies [10,48].

**Fig 3:**
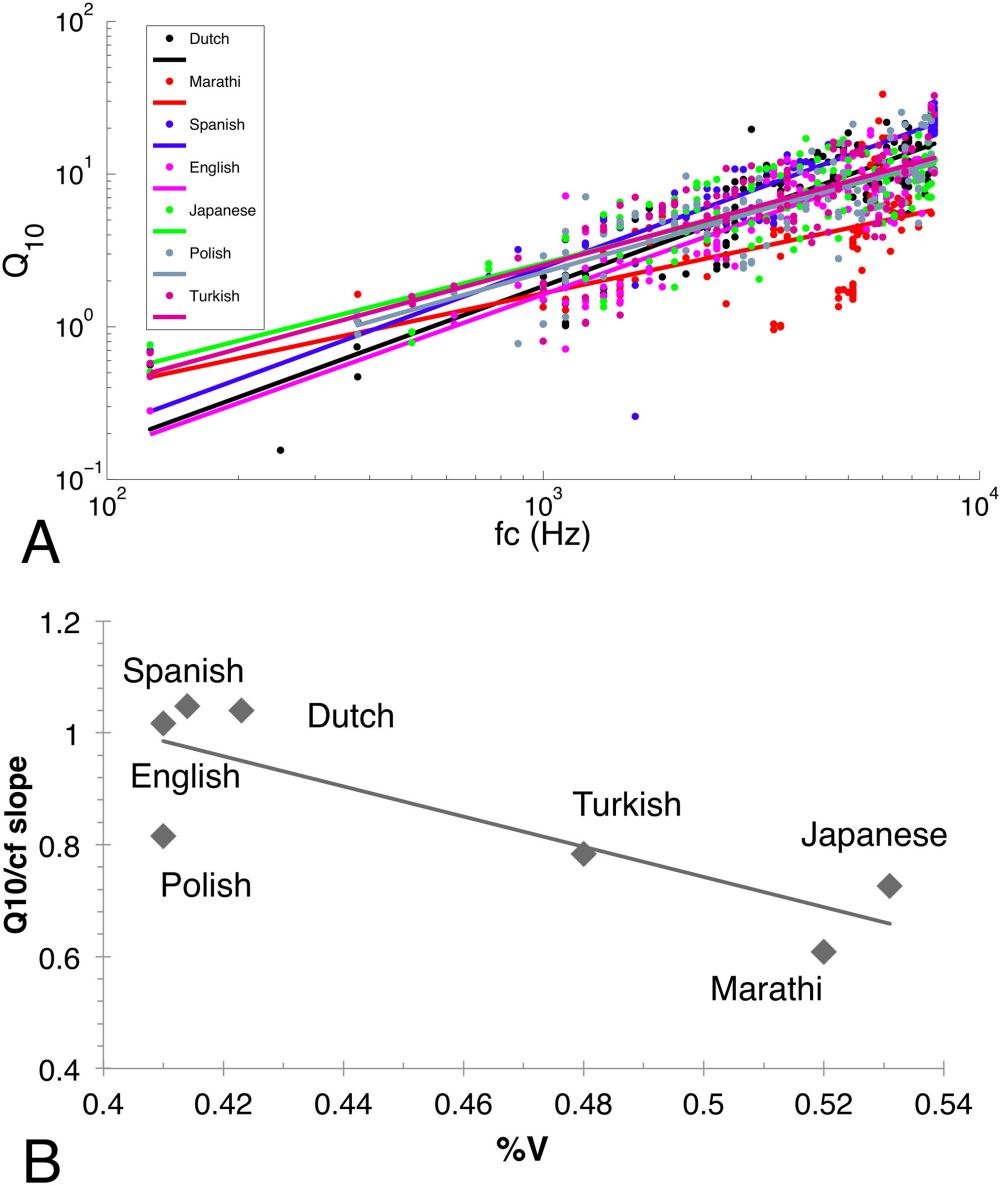
Results of Analysis 1. A. The sharpness of the derived filter populations as a function of center frequency for the seven languages. B. Comparison with %V.

To compare the filter populations obtained for the different languages to one another and to the existing rhythmic measures, we quantified the time-frequency properties of the filter populations, as in [10,20]. For each filter in a population, we obtained its spectrum using a fast Fourier transform (Fig 2). We then calculated its center frequency, bandwidth and sharpness (*Q*_*10*_, center frequency *f*_c_ divided by the bandwidth *Δf*) at a drop of 10dB on either side of the spectral peak. (*Q*_*10*_ cannot be defined if a filter lacks enough depth in its central peak. In our dataset, *Q*_*10*_ could be calculated for most filters in all languages. To compensate for lack of depth, alternatively, we could have calculated sharpness at drops smaller that 10 dB, but in pilot calculations, this resulted in bandwidth values too small for proper spectral analysis.) Sharpness was then plotted against center frequency for all filters in the population for a given language (Fig 3A), and a linear regression fit was obtained. As the slopes (Fig 3A) indicate, filter bandwidth and sharpness increase with center frequency in all languages (for filter bandwidth we obtained $\Delta f\;=\;A{f_{c}}^{1-k}$, with *A* a constant and *k* the slope of the regression lines in Fig 3A). In a linear regression analysis, center frequency *f*_c_ significantly predicted sharpness *Q*_*10*_ for all languages [Dutch: β = 0.889, t(122) = 21.465, p < 0.001, R^2^ = 0.789, English: β = 0.875, t(118) = 19.588, p < 0.001, R^2^ = 0.763, Japanese: β = 0.790, t(112) = 13.656, p < 0.001, R^2^ = 0.621, Marathi: β = 0.563, t(104) = 6.951, p < 0.001, R^2^ = 0.311, Polish: β = 0.813, t(119) = 15.235, p < 0.001, R^2^ = 0.658, Spanish: β = 0.892, t(79) = 17.501, p < 0.001, R^2^ = 0.792, Turkish: β = 0.815, t(109) = 14.659, p < 0.001, R^2^ = 0.660 –degrees of freedom vary across languages, as there were different numbers of filters in the different languages whose spectra were not deep enough to have a well-defined *Q*_*10*_]. This linear relationship between *f*_*c*_ and *Q*_*10*_ implies that, as expected, all languages show a 1/*f* average power spectrum. Importantly, however, the slopes of the linear regression were different across languages. To test for this difference, we have run an analysis of covariance on *Q*_*10*_ as the dependent variable with Language (Dutch / English / Japanese / Polish / Marathi / Turkish / Spanish) as the independent variable and *f*_*c*_ as a covariate. As the previous correlations suggest, *f*_*c*_ had an effect on *Q*_*10*_ (F(1,769) = 837.452, p < 0.001, η_p_^2^ = 0.649). Even more importantly, there was a significant effect of Language on *Q*_*10*_ even after controlling for the effect of *f*_*c*_ (F(6,769) = 29.470, p < 0.001, η_p_^2^ = 0.213). Post hoc pairwise comparisons revealed that Spanish with the steepest slope and Marathi with the lowest slope were different from all other languages (p = 0.001 for all pairwise post hocs for both languages). In addition, English differed from Dutch (p = 0.004), Japanese (p = 0.029) and Turkish (p = 0.004). The other pairwise comparisons were not significant.

To test whether these differences were related to rhythmic differences between the languages, the slopes were compared to previous rhythm analyses [21,27,28]. Interestingly, the slopes show a very high negative correlation (r = -0.84, p = 0.011) with %V (Fig 3B).

### Discussion

We have derived efficient filter populations for seven different languages. We have obtained filters that are localized in time and in frequency for all of the languages, falling in between wavelet-like filters and Fourier-transforms. Furthermore, all filter populations show a scaling relationship between the center frequencies of filters and their bandwidth and sharpness, related to the fact that the average power spectrum for each language is approximately 1/*f* [19]. Importantly, however, we have found that the regression line between center frequency and sharpness has different slopes for different languages, indicating that efficient filters are able to capture cross-linguistic variation in sound patterns.

Furthermore, we have found that this cross-linguistic difference is negatively correlated to a measure of linguistic rhythm, %V, i.e. the proportion of vocalic intervals in the speech signal. The shorter the vocalic intervals in a language, the steeper the slope of the regression line. Since %V and %C add up to 100% [21], the slope of the regression line positively correlates with %C, i.e. the longer the consonantal intervals in a language, the steeper the regression line. In [10], steeper regression lines were associated with animal vocalizations. The negative, rather than positive correlation with %V is therefore unexpected, if vowels resemble animal vocalizations, i.e. if harmonicity plays an important role. Rather, this negative correlation with %V and the concomitant positive correlation with %C imply that acoustic properties of consonants are more relevant, along the lines of [20]. In other words, the properties of the efficient filter populations correlate with speech rhythm, but this correlation may be mediated by acoustic properties other than the simple relative proportion of vowels and consonants, as could have been initially expected on the basis of previous work [10]. Rather, the efficient filter code properties and language rhythm both seem to be correlated with a third factor related to the acoustic properties of consonants.

What are these acoustic properties? If acoustic transience is at play, as suggested by [20], then efficient codes should vary as a function of the types of consonant classes a language has and the relative proportion of these different classes in the speech signal. Work in language typology [39,40,46] shows that these properties are related to the complexity of the different syllable structures a language allows, which in turn is related to the %V and %C values for a given language. Japanese, for instance, has almost exclusively CV syllables (e.g. *Kurisumasu*, the Japanese adaptation of the English word *Christmas*), thus the relative proportion of vowels and consonants is balanced, resulting in relatively high values of %V (above 50%). By contrast, Dutch and English allow complex consonant clusters in syllable onsets and codas, i.e. CCCVCCC (e.g. *springs*).

The current results thus imply that transience might underlie the observed correlation with speech rhythm and might play an important role in determining the statistical structure and hence the properties of the efficient filters of different language. To test this hypothesis, we need to test whether the properties of the efficient codes depend on the consonant classes found in the languages tested. The “sasasa” and “saltanaj” transformations [38] provide an ideal testing ground, as the “saltanaj” transformation preserves consonant class identity, i.e. the degree of transience of a consonant, while the “sasasa” transformation suppresses it. Furthermore, these transformations have been applied to the same Dutch, Japanese, English and Polish sound files that were used in analyses of speech rhythm in previous studies [34,38] and of efficient coding above, in Analysis 1. Therefore, they constitute an optimal ground for comparison with the previous results.

## Analysis 2

In the current analysis, we derived efficient population filters for “sasasa” and “saltanaj” versions of the Dutch, Japanese, English and Polish material from Analysis 1. Since the “saltanaj” resynthesis preserves consonant class identity, and thus transience, we expected this transformation to provide efficient filters with sharpness regression slopes similar to those of the original languages, whereas we predicted the “sasasa” transformation, suppressing the differences between consonant classes, to yield efficient filters with different slopes. Even more specifically, if the claim [20] about the importance of transience alone is correct, the slopes for the “sasasa” versions are expected to be steeper than those of the original or “saltanaj” versions, as “s” is a fricative consonant, and in [20], fricatives have been found to have efficient codes with steep regression slopes. Depending on the nature and frequency of consonants from different consonant classes in a language, the transformations might impact languages to different extents, but the direction of the change (a steeper slope than in the original) should be the same across languages.

### Stimuli

The speech samples consisted of “sasasa” and “saltanaj” versions of the Dutch, English, Japanese and Polish samples used in Analysis 1 and taken originally from [21,34,38]. For Dutch and Japanese, both the “sasasa” and the “saltanaj” resyntheses were available, for English and Polish, only the critical “sasasa” version could be obtained. The “saltanaj” transformation consists of replacing each vowel by an /a/, each fricative by /s/, each liquid by /l/, each occlusive by /t/, each nasal by /n/, and each glide by /j/. The “sasasa” transformation replaces each vowel by an /a/ and each consonant by an /s/, independently of consonant class. The details of the resynthesis are described in [21,34,38]. The sound files had a sampling rate of 16 kHz.

### Algorithm

The ICA algorithm was identical to the one used in Analysis 1.

### Results

**Fig 4:**
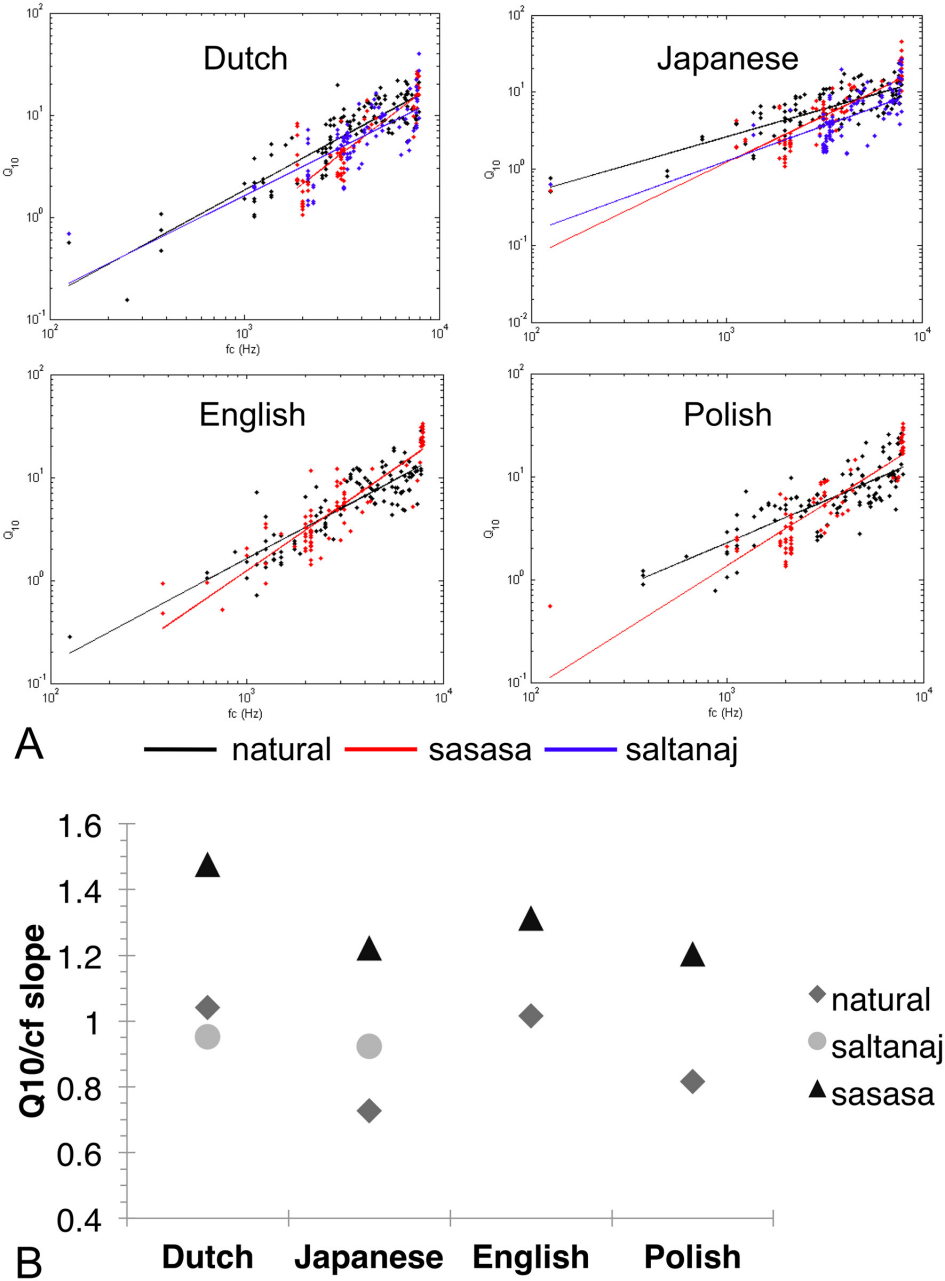
The Results of Analysis 2. A. The sharpness of the derived filter populations as a function of center frequency for Dutch, Japanese, English and Polish natural, saltanaj and sasasa stimuli. B. The values of the slopes as a function of language and transformation.

The ICA algorithm generated a set of efficient filters for each resynthesized version. Overall, the filters have similar shapes to those obtained in Analysis 1, localized in time and frequency. The regression between center frequency and Q_10_ sharpness was calculated in the same way as in Analysis 1 (Fig 4A). Center frequency *f*_c_ significantly predicted sharpness *Q*_*10*_ for all transformed languages [English “sasasa”: β = 0.900, t(86) = 19.180, p < 0.001, R^2^ = 0.808, Dutch “sasasa”: β = 0.871, t(88) = 16.669, p < 0.001, R^2^ = 0.757, Dutch “saltanaj”: β = 0.751, t(105) = 11.637, p < 0.001, R^2^ = 0.559, Polish “sasasa”: β = 0.877, t(86) = 16.959, p < 0.001, R^2^ = 0.767, Japanese “sasasa”: β = 0.845, t(84) = 14.463, p < 0.001, R^2^ = 0.710, Japanese “saltanaj”: β = 0.659, t(108) = 9.107, p < 0.001, R^2^ = 0.429]. We thus observe again a scaling relationship between center frequency and bandwidth for all the resynthesized versions, due to the 1/*f* average power spectrum of the stimuli.

Importantly, however, there are important differences in the regression slopes of the different resynthesized speeches (Fig 4). As we lack the “saltanaj” versions for English and Polish, we ran two ANCOVAs: one comparing the original and the “sasasa” versions in all four languages, and one comparing the original, the “sasasa” and the “saltanaj” versions for Japanese and Dutch. Importantly, for the “sasasa” transformation, which is the crucial manipulation, where we expect differences between the slopes of the original and the resynthesized versions if consonant sub-class identity is relevant, we have data from all four languages tested. While we lack two languages for the “saltanaj” version, the ones we have been able to test, Japanese and Dutch, greatly differ in their speech rhythm, syllable structure and phoneme inventory. Thus, they still constitute a strong test case.

The ANCOVA with factors Language (Japanese / Dutch / English / Polish) and Version (original / “sasasa”) and covariate *f*_*c*_ over Q_10_ as the dependent variable yielded a significant effect for the covariate *f*_*c*_ (F(1,822) = 2041.512, p < 0.001, η_p_^2^ = 0.713) due to the already observed scaling relationship between *f*_*c*_ and Q_10_. It also yielded a significant main effect of Version (F(1,822) = 20.214, p < 0.001, η_p_^2^ = 0.024), as the regression slopes were higher for the “sasasa” versions than for the original ones, as well as a significant Version x Language interaction (F(3,822) = 7.036, p < 0.001, η_p_^2^ = 0.025), since the slope differences between the original and the “sasasa” versions were not the same in all the languages (the difference was greater for Dutch and Japanese than for English and Polish). The main effect of Language wasn’t significant (F(3,822) = 0.980, n.s.).

The ANCOVA with factors Language (Japanese / Dutch) and Version (original / “sasasa” / “saltanaj”) and covariate *f*_*c*_ over Q_10_ as the dependent variable yielded a significant effect for the covariate *f*_*c*_ (F(1,624) = 1109.107, p < 0.001, η_p_^2^ = 0.640) due to the already observed scaling relationship between *f*_*c*_ and Q_10_. The main effect of Version was also significant (F(2,624) = 37.343, p < 0.001, η_p_^2^ = 0.107), because the regression slopes differed across the three versions (for all pairwise post hocs p < 0.05). The main effect of Language was marginally significant (F(1,624) = 3.414, p = 0.065, η_p_^2^ = 0.005), as the slopes for Japanese and Dutch tended to differ overall. The Version x Language interaction (F(2,624) = 9.467, p < 0.001, η_p_^2^ = 0.029) was also significant, since the slope differences between the three versions were not the same in Japanese as in Dutch (the slopes for the “sasasa” transforms were steeper in both languages than for the other two versions, while the “saltanaj” version was very close to the original in Dutch, but somewhat steeper in Japanese).

As predicted, the “saltanaj” versions have slopes closer to those of the original speech stimuli both for Dutch and Japanese. By contrast, the slopes of the “sasasa” versions are much higher in all four languages than those of the original recordings or those of the “saltanaj” versions. Furthermore, while the “sasasa” resynthesis impacts different languages to a different extent, its effect goes in the same direction for every language, and it is sufficient to suppress the overall cross-linguistic differences that were previously observed for the original versions.

### Discussion

The results of Analysis 2 show that the “saltanaj” and “sasasa” manipulations modify the properties of the efficient filters in the predicted way, modifying only slightly and non-systematically the Q_10_ regression slope when consonant class identity (defined by manner of articulation) is maintained (in the “saltanaj” version) and increasing its steepness when consonant class differences are suppressed and replaced by a fricative, i.e. high slope, consonant. This effect has been observed for all four languages. These results thus confirm that consonant class identity is crucial for defining the statistical properties of the speech signal and of its efficient filter population. Consequently, they converge with the proposal regarding the importance of acoustic transience in determining the efficient codes for sound stimuli [20]. Furthermore, while the “sasasa” transformation increased the slopes of the regression lines in all of the language as predicted, its impact was modulated by the specific properties of the languages. This is not unexpected, as languages differ in the number of vowel and consonant sub-classes they have, in the number of phonemes belonging to each class, as well as in the actual acoustic realization of phonemes (e.g. voice onset time for the same consonant tends to be longer in English than, say, in French [53]). It will be interesting in the future to explore exactly how and why the transformations used here affect the signal in each language in exactly the way they do. However, this cross-linguistic modulation of the resynthesis is relatively small compared to the overall change in slope observable in all languages and it does not relate directly to the questions we are asking here. We, therefore, leave the investigation of the specific acoustic details for future research.

Admittedly, the scope of Analysis 2 is limited, mainly due to the limited amount of data available to us from the previously published rhythm studies. Only a subset of the languages from Analysis 1 could be included. Also, the sound files were sampled at 16 kHz, which limits the analyzable frequencies to below 8kHz, which for certain fricatives as produced by female speakers may not cover the full range of relevant frequencies. These limitations notwithstanding, the results of Analysis 2 are consistent with our general hypotheses.

## General Discussion

We have performed two analyses, deriving information theoretically optimal filters for seven rhythmically different languages and their consonant-class preserving and suppressing resyntheses. We have found that all languages exhibit a scaling relationship between the center frequency and the bandwidth of filters within a population, in line with previous results [10] and the general finding that speech, music and some other auditory stimuli have a 1/*f* average power spectrum [19]. In addition to this general similarity, however, we have observed a difference in the sharpness regression slopes of the efficient filter populations for the different languages. This difference correlated negatively with the proportion of vocalic space and positively with the proportion of consonantal space in the speech signals of these languages. Furthermore, the slope values were maintained through a transformation that suppressed consonant identity, but maintained consonant class membership. By contrast, the slope values changed when even consonant membership was neutralized. The direction of this change corresponded to our predictions. All consonants being replaced by an /s/, a fricative consonant with a steep slope, the slopes increased for all languages after the membership-suppressing transformation.

These results suggest that consonants, in particular the distribution of consonants belonging to different consonant classes, play the most important role in determining the statistical properties of the efficient filter populations for different speech stimuli. This, in turn, entails that acoustic transience, the acoustic property that most clearly discriminates consonant classes, underlies important differences in the statistical structures of speech signals from rhythmically different languages [20].

Several points regarding our results deserve further discussion. First, the two phonological properties we considered in our analyses, speech rhythm and consonant inventories are not independent [22,46,47]. Languages with higher %V and thus lower %C values tend to have simpler syllables, allowing no or only simple consonant clusters in the onset and coda positions of syllables, whereas languages with lower %V and thus higher %C values have complex syllables, with heavy consonant clusters in their syllables. This link between rhythm and consonant inventory explains why we found a negative correlation, rather than no correlation at all, between properties of the efficient codes and speech rhythm. Importantly, however, the negative direction of this correlation was contrary to what would have been expected, if the vowel/consonant distinction, and hence harmonicity played a role. In this case, we would have expected a positive correlation between %V and Q_10_ regression slopes. Interestingly, and in confirmation of our conclusion that consonant inventories correlate with rhythmic properties and underlie the statistical structure of speech, a previous study proposing an alternative metric for speech rhythm has arrived to a similar conclusion, starting out from a different approach [30]. In this study, the authors developed an automated way of measuring speech rhythm using a rough estimate of sonority defined directly from the spectrogram of the speech signal. This algorithm successfully reproduced the rhythmic classes, as defined by %V, ΔV and ΔC. Further, by analyzing the distribution of sonorant vs. obstruent segments in the signal across different languages, this study also suggested that rhythmic differences were mostly carried by the less sonorant parts of the signal.

Second, all filter populations showed a scaling relationship between the center frequencies of filters and their bandwidth and sharpness. This is attributable to the fact that the average power spectrum for each language is approximately 1/*f* [19]. From a more general computational perspective, 1/*f* power spectra are an indication of a complex temporal random process [54]. Self-similarity in the spectrum implies an auto-correlation function that is slowly decaying in time: a long-lasting memory random process for which present behavior is strongly affected by the entire history of the system. This is because, according to the Wiener-Kinchin theorem, the autocorrelation function of a random process is given by the Fourier transform of its power spectrum (spectral power density). Using this theorem, it is possible to derive the autocorrelation function of the process (in the time domain) from its power spectrum (in the frequency domain). For pink (1/*f*) noise this results in an autocorrelation function that decays very slowly (logarithmically) with time, i.e. the system has a very long memory, the present state depends strongly on past states. Speech, whereby subsequent linguistic units often predict one another at multiple levels with relatively high probabilities, is exactly this type of process.

Third, the current study investigated the acoustic and statistical properties of the speech signal from a computational point of view. It, therefore, leaves open the question of what the psychophysical, psycholinguistic and neural correlates of these information theoretical mechanisms might be. It has been proposed [20] that the different center frequency—sharpness slopes observed for different phoneme sub-classes might have some neural plausibility, as these mathematically calculated filter properties show similarities to response profiles of different cochlear nuclei in the mammalian auditory system (e.g. multipolar cells in the posteroventral cochlear nucleus have high temporal resolution and a shallow sharpness slope, similarly to the filters derived for stop consonants in [20]). As for speech perception, one study suggests that noise-vocoded speech sounds are better perceived and discriminated if the filters used for the synthesis follow the principles of efficient coding as compared to simple linear filters. While these questions will need to be addressed in future empirical work, here we speculate that the perceptual attunement to the native language which takes place during the first year(s) of life and which is known to involve an attunement to the rhythmic properties [22,35–37] might be paralleled, at the neural level, by an adjustment of the weights of different cochlear filters to best fit the statistical properties of native speech. We are currently testing this hypothesis in our laboratory in brain imaging studies with newborns and young infants.

In this study, we used a time window of approx. 8msec as input samples to the ICA algorithm. Our analysis thus captures auto-correlation between (sub)phonemic units (consonant classes). Languages also different in their sound patterns at larger linguistic units, e.g. in their word- or phrasal level prosodic patterns, in their utterance-level intonation etc. Further research using similar analyses, but with longer samples, will need to investigate whether these suprasegmental differences across languages can also be captured by the principles of efficient coding.

## Conclusion

Our results reveal a significant new link between theoretically derived efficient neural codes and acoustic properties of the speech signal known to be crucial for speech perception and language acquisition. Being a native listener thus involves, among other important abilities, the fine-tuning of the auditory code to the statistics of the native language.
