# Correction: The Efficient Coding of Speech: Cross-Linguistic Differences

**DOI:** 10.1371/journal.pone.0331147

**Published:** 2025-08-28

**Authors:** Ramon Guevara Erra, Judit Gervain

In the Stimuli subsection of the Analysis 1, a reference is omitted from the first sentence of the first paragraph.

The correct sentence is: The speech samples consisted of sentences recorded by female native speakers in Dutch, English, Japanese, Marathi, Polish, Spanish and Turkish, all obtained from previous studies on the prosodic and rhythmic properties of these languages [Dutch, English, Japanese, Polish, Turkish (Nazzi et al., 1998); Marathi [22]; Spanish [23].

The reference is: Nazzi, T., Bertoncini, J., & Mehler, J. (1998). Language discrimination by newborns: Towards an understanding of the role of rhythm. *Journal of Experimental Psychology: Human Perception and Performance, 24*, 756–766.
